# 

**Published:** 1898-01-15

**Authors:** 


					I
tub hospital: ABSOLUTELY PURE, therefore BESTJ Jan. isth, ms
CADBURY'S CADBURY'S
,IT. COCOA 'Mil IPS ir^ OOOOA
The standard of highest purity at present _
attainable,"?Lancet, MO ALKALIES USED,
jlAscoty Wes'tern Infirmary
'RWICH HOSPI TAL
^59^Vol. XXIII. [frw^Pefl Saturday,
Jan. 15th, 1898.
i '
[SubscnptaonTenns,] pRICE TWOPENCE.

				

